# Legal Notes for Institutional Workers

**Published:** 1912-07-27

**Authors:** 


### LEGAL NOTES FOR INSTITUTIONAL WORKERS.

(The Editor will be pleased to answer Legal queries in this column.)

### The Privilege of Medical Witnesses.

It may not be generally known that, although the secrets of the consulting-room are not privileged in England, the Australian Parliament has recently passed an Act protecting them from disclosure. Thus, by the Victoria Evidence Act, 1890, s. 55, it is provided that: "No physician or surgeon shall without the consent of his patient divulge in any civil suit, action or proceeding (unless the sanity of the patient be the matter in dispute) any information which he may have acquired in attending the patient, and which was necessary to enable him to prescribe or act for the patient." The meaning of this enactment was recently considered in a case heard in Australia. An action was brought by an executor to recover the money due on a life policy. The insurance company resisted payment on the ground that the deceased had made untrue statements as to her state of health, and sought to establish their defence by showing that when she signed the policy she was suffering from chronic salpingitis, from which she eventually died. They sought to examine the medical man who had operated upon her in hospital with regard to her condition, but the evidence was objected to and disallowed. The Court held that the section extends to anything which comes to the knowledge of physicians or surgeons with regard to the health or physical condition of the patient, as well as anything said by the patient to him, while the relationship of medical adviser and patient continues provided that it was reasonably necessary for the purpose stated. The judges also expressed the opinion that the prohibition did not cease upon a prescription being given or an operation performed, but that it extended to all the information given until the professional attendance is at an end. They also expressed the view that the operation of the section was not limited to the lifetime of the patient, and left it in doubt whether the executor of a deceased patient could consent to the information being divulged.

### Reasons for the Rule.

The reasons assigned by Mr. Justice Barton for the legislation are well worth reproduction. He points out that: "The mischief for which the common law did not provide was that the confidences reposed by sick people in their medical men were not safe. Communications were only part of such confidences. No matter what the confidence was —howsoever intimate, and in whatsoever manner gained—he who held it was compellable to disclose it in the interests of justice. The range of confidence in matters of bodily health is much wider than in the case of the confessional. A patient has not only symptoms to relate but a body to lay bare as the source of his symptoms. They may be anguish long concealed now first to be discovered by the doctor, or a drug-taking habit which for worlds he would not let anyone but the doctor trace to its cause; or he may have secreted in his room something which, without word spoken or sign made, will tell the doctor when he sees it that which he needs to know. . . . The fact that the medical man was bound to disclose to the world in open Court, whenever so directed by the tribunal, every bit of information he had gained during the professional attendance could not but act as a deterrent, so as to cause patients in many cases to suffer agony and perhaps death rather than divulge fact inexorably kept secret. That dread, whether the fear of exposure and humiliation or the recoil from the infliction of injury or grief on others, was the real mischief which the common law did not provide against if the Legislature saw fit to consider it a mischief, and their opinion is easily traced in the width and the terms they adopted in their provision."

